# Targeting CD33 for acute myeloid leukemia therapy

**DOI:** 10.1186/s12885-021-09116-5

**Published:** 2022-01-03

**Authors:** Jingjing Liu, Jiayin Tong, Haiping Yang

**Affiliations:** Department of Hematology, First Affiliated Hospital of Henan University of Science and Technology, 636 Guanlin Road, Luoyang, Henan 471000 P.R. China

**Keywords:** Acute myeloid leukemia, Immunotherapy, CD33

## Abstract

**Background:**

The aim of this study was to analyze the level of CD33 expression in patients with newly diagnosed AML and determine its correlation with clinical characteristics.

**Methods:**

Samples were collected for analysis from AML patients at diagnosis. We evaluated the level of CD33 expression by flow cytometry analysis of bone marrow. Chi-square or t- tests were used to assess the association between the high and low CD33 expression groups. Survival curves were generated by the Kaplan-Meier and Cox regression model method.

**Results:**

In this study we evaluated the level of CD33 expression in de novo patients diagnosed from November 2013 until January 2019. The mean value of 73.4% was used as the cutoff for the two groups. Statistical analysis revealed that 53 of the 86 (61.2%) AML patients were above the mean. Although there was no statistical significance between CD33 expression level and gene mutation, FLT3 mutation (*P* = 0.002) and NPM1 mutation (*P* = 0.001) were more likely to be seen in the high CD33 group. The overall survival (OS) was worse in the high CD33 group (39.0 m vs. 16.7 m, x^2^ = 13.06, *P* < 0.001). The Cox survival regression display that the CD33 is independent prognostic marker (HR =0.233,*p* = 0.008). Univariate analysis showed that the high expression of CD33 was an unfavorable prognostic factor. Of the 86 patients, CD33-high was closely related to the patients with normal karyotype (x^2^ = 4.891,*P* = 0.027), high white blood cell count (WBC, t = 2.804, *P* = 0.007), and a high ratio of primitive cells (t = 2.851, *P* = 0.005).

**Conclusions:**

These findings provide a strong rationale for targeting CD33 in combination with chemotherapy, which can be considered a promising therapeutic strategy for AML.

**Supplementary Information:**

The online version contains supplementary material available at 10.1186/s12885-021-09116-5.

## Background

In adult patients, acute myeloid leukemia (AML) is a common hematological malignancy. Most of the patients have a poor prognosis. Conventional chemotherapy for AML, including induction and consolidation treatment, is only partially effective. Patients often require bone marrow transplantation and multiple rounds of consolidation therapy. Even with regular chemotherapy, the overall 5-year survival remains below 30% for all patients and is lower for older patients. Most importantly, many patients are refractory to conventional chemotherapy [1, 2]. Thus, we need to develop new methods to improve survival in this fatal form of leukemia. Recently, immunotherapy has been recognized as a new treatment strategy for hematologic malignancies [3, 4]. CAR-T immunotherapy has shown excellent results in ALL (acute lymphocytic leukemia) and lymphoma, and it is possible that patients with acute myeloid leukemia (AML) can also benefit from this treatment strategy [5].

Unlike ALL, AML surface antigens are highly heterogeneous. We need to identify antigens that are specifically expressed in AML as markers for immunotherapy. CD123, CD44, CD174 and CD33 are expressed in hematopoietic stem and progenitor cells, increasing off-target toxicity and killing hematopoietic stem and progenitor cells in immunotherapy [3, 6, 7].

Most efforts of developing monoclonal antibodies or antibody-drug conjugates (ADCs) for AML have focused on targeting CD33 (cluster of differentiation antigen 33). Leukemic blasts and myeloid leukemia-initiating cells express CD33. CD33 does not appear on the surface of primitive stem cells or multipotent progenitor cells. These factors make it a favorable target for immunotherapy of AML [1, 8–10]. There have been a number of reports confirming that CD33 is a feasible target for immunotherapy of AML. Due to the approval of anti-CD33 Mylotarg® (GO, gemtuzumab ozogamicin) in 2000, GO was the first anticancer ADC on the market [8, 10–12]. In our study, we investigated the correlation between the level of CD33 expression in patients with newly diagnosed AML and the prognosis of patients. All data were obtained from 86 newly diagnosed AML patients. Of course, all processes met the ethical standards, and patient consent was obtained. This study provides more persuasive evidence for the immunotherapy of AML.

## Patients and method

### Patients

**Fig. 1:**
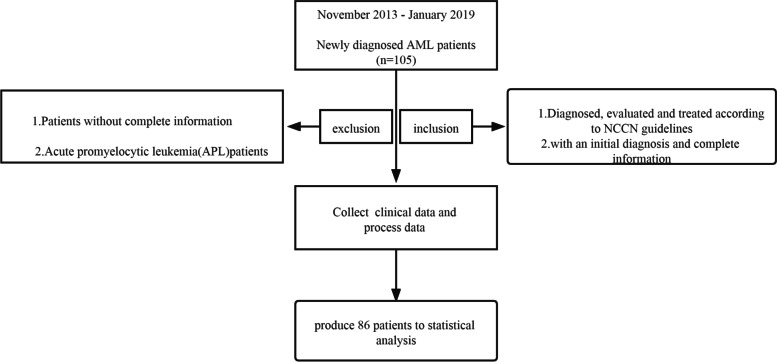
Flow diagram of inclusion criteria used for retrospective study

Between November 2013 and January 2019 in our institution (Department of Hematology, First Affiliated Hospital of Henan University of Science and Technology), 86 patients with an initial diagnosis and complete information were enrolled in the study group(*n* = 86;Fig. 1). All patients were diagnosed, evaluated and treated according to National Comprehensive Cancer Network (NCCN) guidelines [13]. All patients’ records were evaluated retrospectively for the level of CD33 expression in de novo AML patients. Patients were grouped according to expression levels above and under the mean, that is, into high and low level of CD33 expression groups. The association between patient clinical information and CD33 expression was analyzed. Detailed baseline characteristics are shown in Table 1.The molecular information was retrieved from patients’ clinical information rather than newly measured.

**Table 1:** Patients’ characteristics at initial diagnosis

Variables	Low-CD33(*n* = 33)	High-CD33(*n* = 53)	P
Sex(N,M/F)	20/13	34/19	0.742
Age [Y,M (range)]	44 (11–83)	47 (16–86)	0.457
WBC[10^9^/L,M (range)]	16.30 (0.8–145.2)	54.66 (0.9–471.6)	0.007
PLT[10^9^/L,M (range)]	58.71 (7.0–309.0)	58.70 (3.0–275.0)	1.000
primitive cell (%)	46.64 (16.7–81.5)	60.60 (7.5–98.8)	0.005
FAB subtype (N,%)			0.063
M0	0 (0)	1 (1.9)	
M1	0 (0)	4 (7.5)	
M2	27 (81.8)	23 (43.4)	
M4	5 (15.2)	16 (30.2)	
M5	1 (3.0)	9 (17.0)	
Karyotype			0.027
Normal	10 (30.3)	29 (54.7)	
Other	23 (69.7)	24 (45.3)	
Gene			0.114
Mutated	25 (75.8)	47 (88.7)	
Non-Mutated	8 (24.2)	6 (11.3)	
Induction chemotherapy			0.775
CR (N,%)	17 (51.5)	29 (54.7)	
NR (N,%)	16 (48.8)	24 (45.3)	
Relapse or not (N,%)			0.783
Yes	15 (45.5)	21 (39.6)	
No	18 (54.5)	31 (58.5)	
NA		1 (1.9)	

*WBC* the count of white blood cell; *PLT* platelets; *CR* complete remission; *NR* not remission; *YES* referred to relapse; *NO* referred to not relapse; *NA* not available

### Treatments

Induction chemotherapy used standard DA (daunorubicin + cytarabine), IA (nordomycin + cytarabine), MA (mitoxantrone + cytarabine) or CAG with or without the D (arabin cytidine + aclamycin + granulocyte colony stimulating factor with or without decitabine) regimen. Consolidation chemotherapy was given after complete remission (CR), and the regimen referred to induction chemotherapy and usually included cytarabine. For patients with moderate or poor prognosis, allogeneic hematopoietic stem cell transplantation should be performed with appropriate, medium and large dose regimens.

### Methods

A multicolor immunolabeling method was used to determine the expression ratio of CD33. The proportion of CD33 expression was determined by FACS analysis (FACS Navios, Beckman Coulter). as described [14, 15]. Bone marrow chromosome karyotype analysis was performed by the R-banding method. Real-time quantitative PCR (RT–PCR) assay was used to detect fusion genes. Gene mutations were targeted for sequencing by the Illumina sequencing platform. The COSMIC, dbSNP, PolyPhen, SIFT and other databases and software for bioinformatics analysis were applied to determine pathogenic gene mutations [16].

### Efficacy, prognosis evaluation and follow-up

In the retrospective analysis, the risk stratification and the definition of CR, NR, OS, etc. were obtained from the NCCN guidelines of AML [13]. Follow-up was conducted by consulting medical records or via telephone. The follow-up deadline was June 31, 2019.

The probability of OS was calculated according to Kaplan–Meier analysis and compared by the log-rank test using SPSS 20.0.Chose Cox model for survival regression model also using spass 20.0.A *P* < 0.05 was considered significant.

### Statistical analysis

Data were analyzed using SPSS 20.0 (Statistical Program for Social Sciences Version 20.0). Data acquisition was halted on 1st June 2017.

The chi-square test (2-tailed) was used when comparing the categorical variables; a value of *p* ≤ 0.05 was considered statistically significant. Fisher’s exact test (T < 1,or *n* < 40) and t-test (normal distribution and equal variances) were used under the right conditions. The Kaplan–Meier method was used to construct survival curves. Overall survival was calculated from the time of diagnosis to all-cause death. In univariate analyses, log-rank tests were applied to analyze differences in OS between groups. Estimation was limited to the largest survival if it was censored. The Cox model was used as survival regression model to evaluate the independent prognostic role of CD33.Forward: LR method for independent variable screening,including covariates FLT3, NPM1,karyotype, WBC.

## Results

### Patient characteristics

Table 1 summarizes all the characteristics at diagnosis of the 86 patients in the retrospective analysis in the study. Patients were divided into high level of CD33 expression (High-CD33) and low level of CD33 expression (Low-CD33) groups. The distribution of CD33 be reported and described with a boxplot,as shown in supplementary Figure. We found significant differences in the WBC (white blood cell) count, ratio of primitive cells and karyotype between the two groups. These results suggest that CD33 expression is higher in patients with high WBCs, a high proportion of primitive cells and a normal karyotype.

### Gene analysis

The initial evaluation of AML patients is important and it has two prerequisite aims. On the one hand, to characterize the disease process and provide prognostic information, we can base the analysis on factors such as prior toxic exposure, antecedent myelodysplasia, and karyotypic or molecular abnormalities. These factors may impact responsiveness to chemotherapy and the risk of relapse. On the other hand, we assessed the patients’ ability to tolerate chemotherapy by evaluating comorbidities. Both factors are taken into consideration when deciding treatment. Molecular abnormalities play an important role in prognostic stratification. We found molecular abnormalities, including mutant genes and fusion genes, by performing PCR (polymerase chain reaction) and NGS (next-generation sequencing) experiments. Samples for mutation detection were collected from de novo AML patients.

To determine the gene mutation or fusion gene in relation to the level of CD33 expression, we analyzed 86 patients in this retrospective study. In 62 patients (72.1%), molecular abnormalities were detected, which may greatly contribute to the development of AML. Some genes with a high frequency among these gene abnormalities, such as FLT3, NPM1, DNMT3A, IDH1, and CEBPA, are shown in Table 2. The results suggested that more patients with FLT3 mutations (x^2^ = 9.778, *p* = 0.002) and NPM1 mutations (x^2^ = 11.305, *p* = 0.001) presented high levels of CD33 expression. Of the 86 patients, 21 (24.4%) were positive for FLTT3. Among these patients with FLTT3 mutations, there were 19 patients with high CD33 expression and 2 patients with low CD33 expression. Similarly, there were 19 (22.1%) NPM1 mutation patients, of which 18 patients had high levels of CD33 expression.

**Table 2:** Some genes with a high frequency among gene abnormalities

Gene	Low-CD33(*n* = 33)	High-CD33(*n* = 53)	P
FLT3	2	19	0.002
IDH2	2	5	0.880
RUNX1	4	2	0.297
ASXL1	6	3	0.111
KIT	3	4	1.000
DNMT3A	4	16	0.054
IDH1	3	6	1.000
TP53	2	1	0.673
AML1/ETO	3	4	1.000
U2AF1	1	2	1.000
NRAS	2	0	0.144
BCOR	1	2	1.000
CEBPA	3	11	0.154
TET2	2	7	0.490
WT1	3	3	0.863
HOX11	1	0	0.384
NPM1	1	18	0.001

### Survival analysis

**Fig. 2:**
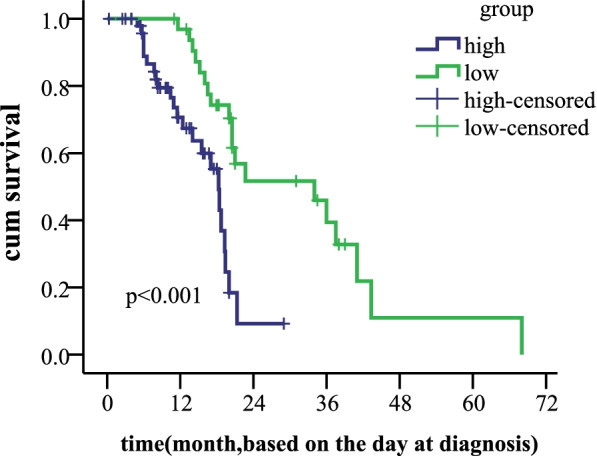
OS of 86 patients with AML based on the expression level of CD33 (low CD33 and high CD33 expression). *P* < 0.001. AML, Acute myeloid leukemia; OS, Overall survival

**Fig. 3:**
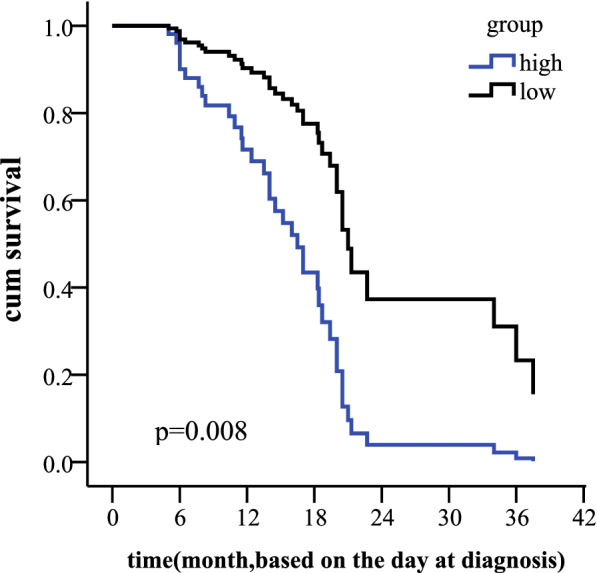
Plot of Cox model survival curves. *P* = 0.002. low, low CD33expression; high,high CD33 expression

The median follow-up time for all patients in the present study, from the day of diagnosis to the last follow-up date, was 16.4 months (range, 0.3–68 months). The OS rates of patients in the low CD33 expression and high CD33 expression groups were 67.5 and 42.9%, respectively (log rank = 7.385, *P* = 0.007). The results are shown in the Fig. 2. The Cox survival regression display that the hazard ratio estimate is HR =0.233,*p* = 0.008(95% confidence interval (CI):0.080–0.679). The hazard ratios (HR) and 95% CI in the final model are shown in the Table 3.The result show that CD33 can be independent prognostic marker in AML,as shown in Fig. 3.

**Table 3:** Variables in the Equation

	B	SE	Wald	df	Sig.	Exp(B)	95.0% CI for Exp(B)	
							Lower	Upper
group	−1.455	.545	7.134	1	.008	.233	.080	.679
FLT3	.497	.396	1.575	1	.209	1.645	.756	3.577
NPM1	.275	.423	.423	1	.515	.760	.332	1.740
karyotype	.303	.321	.890	1	.346	1.354	.721	2.541
WBC	.001	.003	.251	1	.617	1.001	.996	1.007

*WBC* the count of white blood cell; *FLT3* FMS-like tyrosine kinase3; *NPM1* nucleophosmin 1

## Discussion

Acute myeloid leukemia (AML) is a heterogeneous hematological malignancy characterized by the proliferation of leukemia-initiating cells, and the majority of cases have a poor prognosis. It is the most common form of acute leukemia among adults, with the largest number of deaths due to leukemia each year. The median age at diagnosis is 67 years, and 54% of patients are diagnosed when they are 65 years or older (approximately one-third are diagnosed as ≥75 years) [13].

Considerable progress has been made in anticancer treatments and breakthroughs in immunotherapies, but conventional AML chemotherapy, including induction and consolidation therapy, has not changed significantly. This highlights the need to develop new ways to improve prognosis, especially refractory and relapsed AML (R-R AML) [17, 18].

Allogeneic hematopoietic stem cell transplantation (allo-HSCT) may remain the sole curative option. Therefore, immunotherapies, such as ADC, CAR-T and GO, bring promise and challenge the traditional regimens given to many R-R AML patients [9, 11, 19].

Although immunotherapy has been widely accepted as an effective strategy in the field of hematological malignancies, the lack of high specificity of target antigens and the heterogeneity of AML has led to slow progress in treating AML with similar strategies. Therefore, the challenge for us is to determine an antigen expressed specifically on the surface of leukemia cells in AML [4].

In this study, the results showed that the expression level of CD33, an independent risk factor affecting prognosis, was related to the OS of patients. At diagnosis, AML patients with a higher count of white blood cells, a higher percentage of primitive cells, and normal karyotypes had higher levels of CD33 expression. Patients with FLT3 and NPM1 mutations had higher expression of CD33. These positive results suggested that patients would most likely benefit from CD33 targeted therapy when de novo AML patients had the abovementioned clinical characteristics. In our opinion, taking into account all the available data can help improve the prognosis and survival of AML patients. Limitations of this study include the sample size and grouping the patients according to more detailed characteristics would be beneficial for analysis.

Of course, more research is required, preferably prospective clinical data, to optimize the regimen of targeting CD33 for AML. More clinical features or other factors need to be identified to help us choose CD33 as a targeted therapy for AML or in combination with chemotherapy.

Patients with FLT3 and NPM1 mutations had higher expression of CD33, which was consistent with the published literature [20]. Another study reported higher CD33 expression in AMLs with NPM1 mutation,which is confirmed by our findings [21].However,this conclusion needs to be supported by more clinical data. If possible, follow up the levels of cell surface expression of CD33 in AML patients with minimal residual disease based on positivity for NPM1 mut. Perhaps we can operate laboratory research to reveals deeper connections between the level of CD33 and NPM1-mutation. We can propose that CD33 is a target for AML immunotherapy according to our findings [22].

Targeting CD33 appears to be a suitable alternative in patients who lack hematologic stem cell donors. Ongoing efforts are needed to optimize the application to enhance therapeutic effects and decrease injury, including cytotoxicity and economic losses. To investigate the possibility of immune-based therapies beyond stem cell transplantation to treat hematologic malignancies and recommend targeting CD33 therapy for more extended list treatments, future research should focus on studies with higher quality parameters, such as larger sample sizes, randomized studies and prospective studies.

Responsive biomarkers will enable us to select patients who are more likely to benefit from immune checkpoints and monoclonal-based therapies. Exploiting the true potential of immune agents in AML requires excellently designed clinical trials. Trials are ongoing and will guide further development of immune agents [17, 22–25].

## Conclusion

According to these results in the retrospective study, we have reason to believe that CD33 can serves as a promising target of immunotherapy for AML.

## Supplementary Information

**Additional file 1.** Supplementary Figure. The distribution of CD33.

## Data Availability

The datasets used or analysed during the current study are available from the corresponding author on reasonable request.

## Abbreviations

CD: Cluster of differentiation antigen
AML: Acute myeloid leukemia
CAR-T: Chimeric antigen receptor T cell immunotherapy
ALL: Acute lymphocytic leukemia
FLT3: FMS-like tyrosine kinase 3
NPM1: Nucleophosmin 1
ADC: Antibody-drug conjugates
GO: Gemtuzumab ozogamicin
NCCN: Comprehensive Cancer Network
DA: Daunorubicin + cytarabine
IA: Nordomycin + cytarabine
MA: Mitoxantrone + cytarabine
CAG: Arabin cytidine + aclamycin + granulocyte colony stimulating factor
CR: Complete remission
NR: Non remission
OS: Overall survival
PCR: Polymerase chain reaction
RT–PCR: Real-time quantitative polymerase chain reaction
NGS: Next-generation sequencing
WBC: White blood cell
DNMT3A: DNA methyltransferases 3A
IDH1: Isocitrate dehydrogenase 1
CEBPA: CCAAT/enhancer binding protein alpha
allo-HSCT: Allogeneic hematopoietic stem cell transplantation
R-R AML: Refractory-relapse acute myeloid leukemia
LR: Likelihood ratio
CI: Confidence interval
HR: Hazard ratios
