# Supplementary figures and images for: Targeting CD33 for acute myeloid leukemia therapy
Source: BMC Cancer. 2022 Jan 3;22:24. doi: 10.1186/s12885-021-09116-5 (PMC8722076; doi:10.1186/s12885-021-09116-5)

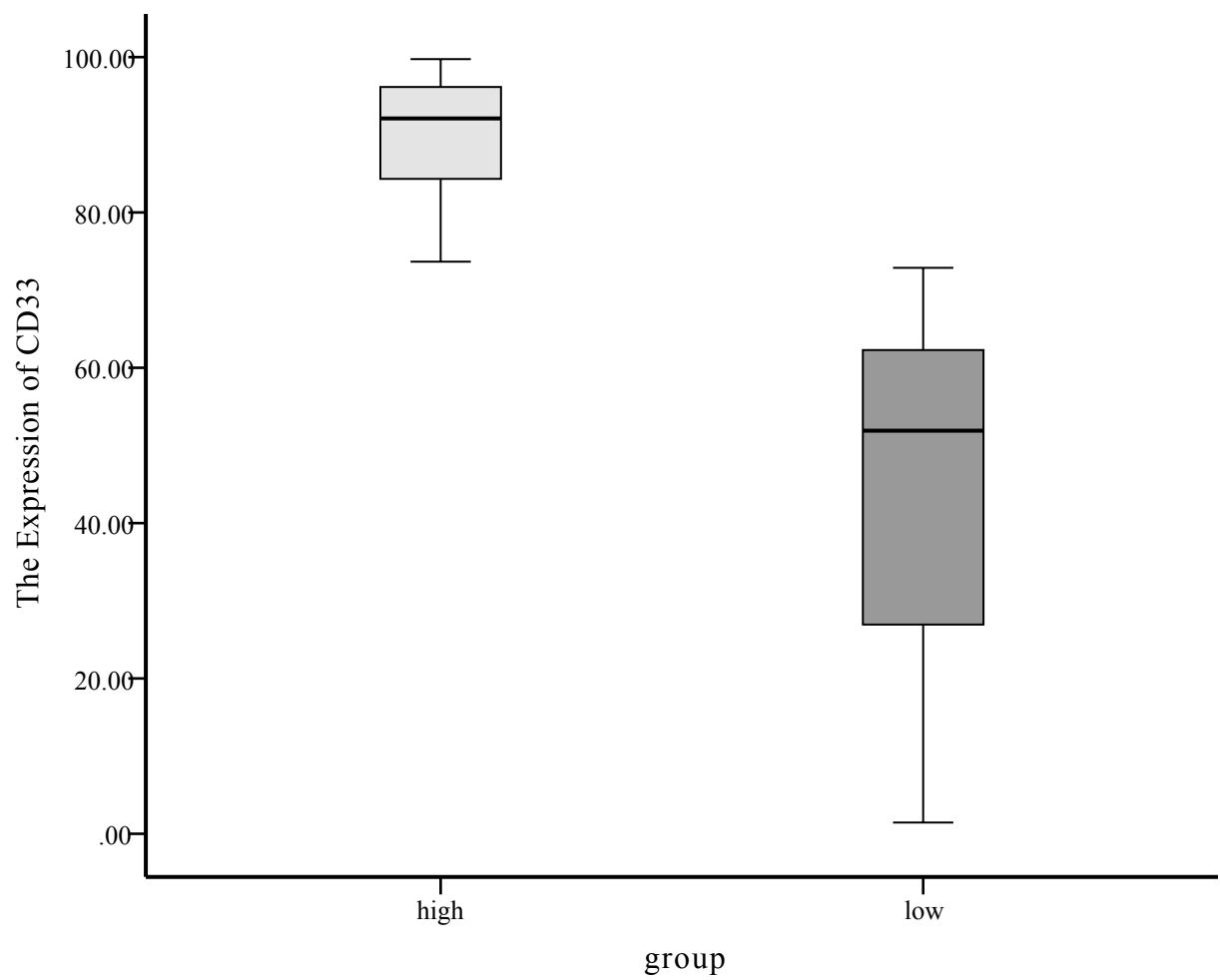

Supplement: Supplementary file 1 — Additional file 1. Supplementary Figure. The distribution of CD33. [file 12885_2021_9116_MOESM1_ESM.pdf]
